# Supplementary material for: Correlations between the Composition of the Bovine Microbiota and Vitamin B12 Abundance
Source: mSystems. 2020 Mar 3;5(2):e00107-20. doi: 10.1128/mSystems.00107-20 (PMC7055655; doi:10.1128/mSystems.00107-20)
Supplement: TABLE S1 [file mSystems.00107-20-st001.docx]

Table S1- Concentration of Vitamin B12 Measured in Each Sample

| **Sample Number** | **Plasma** | **Milk** | **Rumen** | **Feces** |
| --- | --- | --- | --- | --- |
|  | **(pg/ml)** | **(pg/g)** | **(ng/g)** | **(ng/g)** |
| **1** | 196.32 |  | 493.25 | 321.73 |
| **2** | 305.23 | 4511.78 | 1003.26 | 920.31 |
| **4** | 178.19 | 2185.88 | 856.29 | 871.59 |
| **5** | 138.74 | 4773.86 | 669.75 | 564.77 |
| **6** | 119.74 | 2988.33 | 569.43 | 519.63 |
| **7** | 169.82 | 2180.72 | 777.93 | 668.13 |
| **8** | 185.52 | 2754.61 | 811.45 | 565.75 |
| **9** | 266.93 |  | 481.73 | 279.18 |
| **10** | 1051.86 |  | 689.29 | 584.99 |
| **11** | 237.85 | 4789.97 | 544.98 | 399.90 |
| **12** | 127.35 | 8147.37 | 455.27 | 568.28 |
| **13** | 189.90 | 4578.72 | 467.69 | 667.71 |
| **14** | 204.49 | 1876.59 | 734.65 | 588.69 |
| **15** | 186.42 | 1835.92 | 554.11 | 622.09 |
| **16** | 118.75 | 1559.99 | 682.00 | 471.52 |
| **17** | 179.35 | 7310.14 | 660.55 | 574.92 |
| **18** | 267.51 | 4433.29 | 675.70 | 531.42 |
| **19** | 171.14 | 3207.47 | 563.83 | 460.70 |
| **20** | 181.60 |  | 682.66 | 393.28 |
| **21** | 177.65 |  | 322.25 | 233.94 |
| **22** | 148.05 |  | 410.88 | 351.72 |
| **23** | 159.19 |  | 401.14 | 248.46 |
| **24** | 187.75 | 3419.07 | 676.49 | 637.37 |
| **25** | 415.45 | 2448.58 | 768.40 | 506.69 |
| **26** | 271.33 | 2430.52 | 497.45 | 471.26 |
| **27** | 136.60 | 2309.28 | 545.16 | 533.94 |
| **28** | 208.60 | 3029.44 | 697.94 | 832.83 |
| **29** | 118.32 | 2221.21 | 734.30 | 478.69 |
| **30** | 312.57 | 2596.95 | 701.58 | 712.86 |
| **31** | 251.19 | 2777.76 | 405.37 | 550.72 |
| **32** | 127.93 | 3223.44 | 617.85 | 360.87 |
| **33** | 213.35 | 3086.83 | 810.28 | 628.59 |
| **34** | 151.69 | 1999.71 | 809.22 | 598.92 |
| **35** | 274.34 | 4105.35 | 784.87 | 670.40 |
| **36** | 253.05 | 3037.40 | 421.63 | 786.16 |
| **37** | 277.80 | 2649.78 | 519.61 | 660.45 |
| **38** | 238.00 | 4358.62 | 630.03 | 503.79 |
| **39** | 201.27 | 5596.04 | 658.75 | 527.87 |
| **40** | 176.19 | 2927.93 | 650.17 | 422.40 |
| **41** | 350.53 | 7590.09 | 587.02 | 855.42 |
| **42** | 129.01 | 2557.65 | 500.06 | 471.75 |
| **43** | 207.26 |  | 684.63 | 226.10 |
| **44** | 250.99 |  | 271.25 | 175.64 |
| **45** | 159.24 |  | 450.74 | 251.08 |
| **46** | 189.71 |  | 297.98 | 253.31 |
| **47** | 188.65 |  | 501.20 | 310.20 |
| **48** | 196.66 | 2981.82 | 754.93 | 478.21 |
| **49** | 122.73 | 3069.63 | 699.89 | 556.37 |
| **52** | 238.93 | 2681.25 | 605.93 | 766.47 |
| **53** | 249.71 |  | 162.80 | 394.84 |
| **54** | 3111.19 |  | 199.32 | 334.63 |
| **57** | 296.96 |  | 670.26 | 478.28 |
| **58** | 280.87 | 4365.26 | 735.89 | 766.72 |
| **59** | 231.50 | 5225.96 | 1021.39 | 684.12 |
| **60** | 271.79 | 4024.04 | 755.18 | 599.63 |
| **61** | 247.63 | 1496.35 | 812.62 | 421.21 |
| **62** | 399.90 | 4437.24 | 891.00 | 566.28 |
| **63** | 365.39 | 5577.74 | 754.31 | 486.67 |
| **64** | 240.36 | 4355.95 | 1006.62 | 613.34 |
| **65** | 363.96 | 6163.60 | 839.93 | 579.39 |
| **66** | 269.37 | 6079.39 | 939.79 | 751.74 |
| **67** | 228.01 | 5276.06 | 412.71 | 504.78 |
| **68** | 173.17 | 10612.13 | 906.40 | 578.09 |
| **69** | 144.33 | 5999.48 | 578.10 | 340.16 |
| **70** | 239.39 | 8191.46 | 607.54 | 496.60 |
| **71** | 166.91 | 4754.08 | 536.33 | 520.74 |
| **72** | 286.77 | 3688.80 | 594.53 | 536.38 |
| **73** | 169.22 | 5868.98 | 446.61 | 662.16 |
| **74** | 253.85 | 3250.69 | 500.53 | 800.71 |
| **75** | 219.71 | 3443.87 | 443.05 | 564.95 |
| **76** | 189.58 | 5092.48 | 383.20 | 536.50 |
| **77** | 242.55 | 3193.21 | 525.60 | 815.82 |
| **78** | 115.76 | 3045.83 | 621.57 | 618.91 |
| **79** | 242.48 | 2645.11 | 616.97 | 590.07 |
| **80** | 212.72 | 2325.23 | 382.25 | 663.07 |
| **81** | 198.71 | 4300.76 | 300.09 | 522.00 |
| **82** | 142.77 | 5217.17 | 331.02 | 568.38 |
| **83** | 142.48 | 1998.49 | 776.77 | 714.24 |
| **84** | 196.60 | 3274.94 | 495.85 | 538.34 |
| **85** | 139.40 | 3438.73 | 470.95 | 564.51 |
| **86** | 234.70 |  | 325.33 | 307.32 |
| **87** | 1007.96 |  | 909.44 | 294.49 |
| **88** | 215.60 |  | 398.55 | 356.80 |
| **89** | 119.72 | 2099.72 | 804.81 | 834.44 |
| **90** | 163.76 | 1208.93 | 795.72 | 705.66 |
| **91** | 179.92 | 4431.40 | 996.80 | 613.40 |
| **92** | 145.18 | 2078.88 | 623.50 | 661.22 |
| **93** | 182.40 | 1754.63 | 604.55 | 555.33 |
| **94** | 118.69 | 1852.23 | 668.42 | 578.194 |
| **95** | 264.35 | 3896.92 | 534.98 | 481.7916 |
| **96** | 199.70 | 1719.41 | 800.13 | 796.3379 |
| **97** | 181.85 | 2705.69 | 545.55 | 794.5158 |
