# Supplementary material for: Correlations between the Composition of the Bovine Microbiota and Vitamin B12 Abundance
Source: mSystems. 2020 Mar 3;5(2):e00107-20. doi: 10.1128/mSystems.00107-20 (PMC7055655; doi:10.1128/mSystems.00107-20)
Supplement: TABLE S2 [file mSystems.00107-20-st002.docx]

Table S2 – Samples used to analyse differences in the rumen microbiome based on vitamin B12 concentration

|  | High Vitamin B12 | Low Vitamin B12 |
| --- | --- | --- |
| Samples used for Analysis | R2, R4, R7, R8, R25, R33, R34, R35, R48, R58, R59, R60, R61, R62, R63, R64, R66, R89, R90, R91, R96 | R11, R12, R13, R15, R19, R26, R27, R31, R36, R37, R42, R67, R71, R73, R74, R75, R76, R77, R80, R81, R82, R84, R85, R95, R97 |
