# Supplementary material for: Correlations between the Composition of the Bovine Microbiota and Vitamin B12 Abundance
Source: mSystems. 2020 Mar 3;5(2):e00107-20. doi: 10.1128/mSystems.00107-20 (PMC7055655; doi:10.1128/mSystems.00107-20)
Supplement: TABLE S3 [file mSystems.00107-20-st003.docx]

Table S3- PERMANOVA analysis of Bray-Curtis Distance using Adonis

|  | F-value | R-squared | P-value |
| --- | --- | --- | --- |
| PERMANOVA, bray-curtis distance, Vitamin B12 Group | 9.5016 | 0.1712 | <0.001 |
| PERMANOVA, bray-curtis distance, Diet | 8.0209 | 0.14848 | <0.002 |
| PERMANOVA, bray-curtis distance, Vitamin B12 Group, Diet | 10.076 | 0.1712 | <0.001 |
| PERMANOVA, bray-curtis distance, Diet, Vitamin B12 Group | 8.7388 | 0.14848 | <0.001 |
