# Supplementary material for: Correlations between the Composition of the Bovine Microbiota and Vitamin B12 Abundance
Source: mSystems. 2020 Mar 3;5(2):e00107-20. doi: 10.1128/mSystems.00107-20 (PMC7055655; doi:10.1128/mSystems.00107-20)
Supplement: TABLE S4 [file mSystems.00107-20-st004.docx]

Table S4 – LEfSe values for top 50 bacterial taxa at the genus level correlated to vitamin B12 concentration in the rumen*

| **Bacteria Genus** | **P-values** | **FDR** | **High** | **Low** | **LDAscore** |
| --- | --- | --- | --- | --- | --- |
| Prevotella_7 | 1.92E-07 | 4.09E-05 | 373.52 | 7.88 | 2.26 |
| Ruminiclostridium_9 | 1.26E-05 | 0.0013413 | 1.7391 | 16.2 | -0.915 |
| Succinimonas | 4.73E-05 | 0.0033547 | 3.5217 | 6.96 | -0.434 |
| Bacteroidetes_BD2_2_ge | 0.00012835 | 0.0050199 | 3.4783 | 9.8 | -0.619 |
| **Butyrivibrio_2 | 0.00017988 | 0.0050199 | 218.48 | 383.16 | -1.92 |
| **SuccinivibrionaceaeUCG_002 | 0.00019222 | 0.0050199 | 58.913 | 213.32 | -1.89 |
| Lachnoclostridium_10 | 0.00020057 | 0.0050199 | 11.957 | 22.68 | -0.804 |
| Pseudobutyrivibrio | 0.00021177 | 0.0050199 | 86.609 | 171.48 | -1.64 |
| Lachnospiraceae_ND3007_group | 0.00023472 | 0.0050199 | 9.2174 | 20.68 | -0.828 |
| Lachnospiraceae_FCS020_group | 0.00024728 | 0.0050199 | 12.522 | 30.12 | -0.991 |
| Ruminococcus_1 | 0.00025924 | 0.0050199 | 233.22 | 326.4 | -1.68 |
| Blautia | 0.00045723 | 0.0069912 | 9.1304 | 26.12 | -0.977 |
| Lachnospiraceae_XPB1014_group | 0.00048572 | 0.0069912 | 42.696 | 93.84 | -1.42 |
| F082_ge | 0.00048676 | 0.0069912 | 123.52 | 209.64 | -1.64 |
| Shuttleworthia | 0.00052277 | 0.0069912 | 69.348 | 32.8 | 1.28 |
| Succinivibrionaceae_UCG_001 | 0.00052516 | 0.0069912 | 5200.2 | 1466.7 | 3.27 |
| p_251_o5_ge | 0.00057796 | 0.0072415 | 4.5652 | 53.28 | -1.4 |
| Bacteroidales_unclassified | 0.00079242 | 0.009377 | 23.913 | 66.16 | -1.34 |
| Clostridiales_unclassified | 0.00088879 | 0.0099638 | 39.565 | 71.36 | -1.23 |
| Lachnospiraceae_AC2044_group | 0.00095657 | 0.010056 | 37.957 | 67.68 | -1.2 |
| probable_genus_10 | 0.00099144 | 0.010056 | 36.957 | 67.04 | -1.21 |
| Lachnospiraceae_UCG_002 | 0.001185 | 0.011469 | 4.7826 | 8.88 | -0.484 |
| Rikenellaceae_RC9_gut_group | 0.0012385 | 0.011469 | 271.39 | 424.04 | -1.89 |
| ** Fibrobacter | 0.0015356 | 0.013629 | 1321.7 | 2102.6 | -2.59 |
| Pedosphaeraceae_ge | 0.0017038 | 0.014516 | 0 | 2.44 | -0.346 |
| Bacteroidia_unclassified | 0.0019451 | 0.015934 | 32.696 | 56.32 | -1.11 |
| **Christensenellaceae_R7group | 0.0023342 | 0.018414 | 690.96 | 1097 | -2.31 |
| Papillibacter | 0.0028429 | 0.02081 | 14.174 | 25.68 | -0.829 |
| Lachnospiraceae_NK3A20_group | 0.0028625 | 0.02081 | 729.96 | 993.72 | -2.12 |
| Lachnospiraceae_UCG_008 | 0.002931 | 0.02081 | 23.739 | 33.08 | -0.754 |
| Ruminococcaceae_unclassified | 0.003155 | 0.021678 | 25 | 42.96 | -0.999 |
| Flexilinea | 0.0032655 | 0.021736 | 22.261 | 40.4 | -1 |
| vadinBE97_ge | 0.0033769 | 0.021796 | 3.3478 | 9.24 | -0.596 |
| Bacteroidales_UCG_001_ge | 0.0038557 | 0.022736 | 43.217 | 79.8 | -1.29 |
| Ruminobacter | 0.0039645 | 0.022736 | 29.304 | 65.24 | -1.28 |
| Ruminococcaceae_UCG_010 | 0.0039842 | 0.022736 | 41.043 | 71.8 | -1.21 |
| Saccharofermentans | 0.0039862 | 0.022736 | 172.61 | 256.84 | -1.63 |
| WCHB1_41_ge | 0.0041176 | 0.022736 | 102.57 | 178.8 | -1.59 |
| Christensenellaceae_unclassified | 0.0041629 | 0.022736 | 5 | 10 | -0.544 |
| Ruminiclostridium_6 | 0.0054277 | 0.028902 | 6.8261 | 11.2 | -0.503 |
| **Prevotella_1 | 0.0068594 | 0.035635 | 2948.9 | 3726.3 | -2.59 |
| Ruminococcaceae_UCG_005 | 0.010157 | 0.051512 | 45.391 | 67 | -1.07 |
| Acetobacter | 0.010677 | 0.052887 | 2.6957 | 0.64 | 0.307 |
| uncultured | 0.011459 | 0.055473 | 105.04 | 170.36 | -1.53 |
| Bacteroidales_BS11_gut_group_ge | 0.012081 | 0.057181 | 14.783 | 25.88 | -0.816 |
| Oribacterium | 0.012825 | 0.059387 | 67.348 | 25.56 | 1.34 |
| Pseudoscardovia | 0.014982 | 0.067898 | 1.1304 | 0 | 0.195 |
| Prevotella_9 | 0.015358 | 0.06815 | 9.087 | 1.76 | 0.669 |
| Candidatus_Endomicrobium | 0.016166 | 0.070271 | 2.087 | 3.6 | -0.245 |

* Statistically significant correlations are displayed in orange.

** LDA score > 2 (or -2)
