# Supplementary material for: Correlations between the Composition of the Bovine Microbiota and Vitamin B12 Abundance
Source: mSystems. 2020 Mar 3;5(2):e00107-20. doi: 10.1128/mSystems.00107-20 (PMC7055655; doi:10.1128/mSystems.00107-20)
Supplement: TABLE S5 [file mSystems.00107-20-st005.docx]

Table S5 – Samples used to analyse differences in the fecal microbiome based on vitamin B12 concentration

|  | High Vitamin B12 | Low Vitamin B12 |
| --- | --- | --- |
| Samples used for Analysis | F6, F11, F16, F18, F19, F25, F26, F27, F29, F32, F38, F39, F40 F42, F48, F61, F63, F67, F69, F70, F71, F72, F76, F81, F95, | F2, F4, F7, F13, F24, F28, F30, F35, F36, F37, F41, F52, F58, F59, F66, F73, F74, F77, F80, F89, F90, F92, F96, F97 |
