# Supplementary material for: Correlations between the Composition of the Bovine Microbiota and Vitamin B12 Abundance
Source: mSystems. 2020 Mar 3;5(2):e00107-20. doi: 10.1128/mSystems.00107-20 (PMC7055655; doi:10.1128/mSystems.00107-20)
Supplement: TABLE S6 [file mSystems.00107-20-st006.docx]

Table S6 – LEfSe values for top 50 bacterial taxa at the genus level correlated to vitamin B12 concentration in the rumen*

| Bacteria Genus | P- values | FDR | High | Low | LDA score |
| --- | --- | --- | --- | --- | --- |
| Flexilinea | 0.012551 | 0.53864 | 1.6957 | 0.04 | 0.262 |
| p_1088_a5_gut_group | 0.014475 | 0.53864 | 3.3043 | 2.48 | 0.15 |
| Oligosphaeraceae_unclassified | 0.014982 | 0.53864 | 0.78261 | 0 | 0.143 |
| Defluviitaleaceae_unclassified | 0.015127 | 0.53864 | 0.30435 | 0.04 | 0.0539 |
| Victivallales_unclassified | 0.017799 | 0.53864 | 4.4783 | 2.12 | 0.338 |
| Tyzzerella_3 | 0.018986 | 0.53864 | 0.56522 | 0.24 | 0.0654 |
| Moryella | 0.019184 | 0.53864 | 0.47826 | 0.08 | 0.0789 |
| Ruminococcaceae_unclassified | 0.020231 | 0.53864 | 230.91 | 179.24 | 1.43 |
| Victivallaceae_unclassified | 0.024852 | 0.58817 | 5.6957 | 1.4 | 0.498 |
| Succinivibrionaceae_UCG_002 | 0.031357 | 0.61067 | 3.3913 | 0 | 0.431 |
| Alphaproteobacteria_unclassified | 0.034572 | 0.61067 | 1.3043 | 2.68 | -0.227 |
| Erysipelotrichaceae_unclassified | 0.038247 | 0.61067 | 4.1739 | 2.44 | 0.271 |
| GWE2_31_10 | 0.039825 | 0.61067 | 24.087 | 13.28 | 0.806 |
| vadinBE97_ge | 0.042839 | 0.61067 | 16.348 | 4.84 | 0.83 |
| Elusimicrobium | 0.051465 | 0.61067 | 2.9565 | 1.12 | 0.283 |
| Ruminococcaceae_UCG_007 | 0.054105 | 0.61067 | 0.65217 | 0.16 | 0.0955 |
| Akkermansia | 0.058857 | 0.61067 | 25.826 | 49.56 | -1.11 |
| FD2005 | 0.059264 | 0.61067 | 0.6087 | 0.04 | 0.109 |
| Christensenellaceae_unclassified | 0.064968 | 0.61067 | 0.13043 | 0 | 0.0274 |
| Desulfovibrio | 0.064968 | 0.61067 | 0.13043 | 0 | 0.0274 |
| Oscillibacter | 0.070752 | 0.61067 | 52.435 | 39.64 | 0.869 |
| Pseudobutyrivibrio | 0.074591 | 0.61067 | 1.5217 | 0.52 | 0.176 |
| Actinomycetaceae_unclassified | 0.078155 | 0.61067 | 0.26087 | 0.08 | 0.0376 |
| Butyrivibrio_2 | 0.084178 | 0.61067 | 0.91304 | 0.64 | 0.0556 |
| Slackia | 0.089686 | 0.61067 | 0 | 0.16 | -0.0334 |
| Acinetobacter | 0.089686 | 0.61067 | 0 | 0.2 | -0.0414 |
| Prevotellaceae_YAB2003_group | 0.092355 | 0.61067 | 3.8696 | 2.04 | 0.282 |
| Lachnospiraceae_UCG_010 | 0.092842 | 0.61067 | 2.087 | 3.72 | -0.259 |
| Ruminiclostridium_1 | 0.095373 | 0.61067 | 0.6087 | 0.4 | 0.0431 |
| Ruminococcaceae_UCG_009 | 0.10506 | 0.61067 | 73.913 | 62.36 | 0.831 |
| WCHB1_41_ge | 0.10518 | 0.61067 | 156.09 | 105.52 | 1.42 |
| Staphylococcus | 0.10814 | 0.61067 | 0.47826 | 0.4 | 0.0167 |
| Sutterella | 0.11424 | 0.61067 | 6.1739 | 8.16 | -0.3 |
| Ruminococcus_1 | 0.11426 | 0.61067 | 65.391 | 47.84 | 0.99 |
| Paludibacteraceae_unclassified | 0.11674 | 0.61067 | 92.391 | 160 | -1.54 |
| Dorea | 0.11882 | 0.61067 | 24.217 | 31.24 | -0.654 |
| Victivallaceae_ge | 0.1291 | 0.61067 | 43.348 | 33.84 | 0.76 |
| Candidatus_Soleaferrea | 0.13066 | 0.61067 | 1.4348 | 4 | -0.358 |
| Ruminococcaceae_UCG_002 | 0.13153 | 0.61067 | 34.739 | 28.12 | 0.634 |
| Absconditabacteriales_(SR1)_ge | 0.13613 | 0.61067 | 0.086957 | 0 | 0.0185 |
| horsej_a03 | 0.13622 | 0.61067 | 0.13043 | 0 | 0.0274 |
| Synergistes | 0.13622 | 0.61067 | 0.13043 | 0 | 0.0274 |
| Succinimonas | 0.13622 | 0.61067 | 0.30435 | 0 | 0.0615 |
| Kurthia | 0.13622 | 0.61067 | 0.43478 | 0 | 0.0854 |
| Rhodospirillales_unclassified | 0.14509 | 0.61067 | 1.2174 | 1.88 | -0.124 |
| DSSD61 | 0.17033 | 0.61067 | 0 | 0.08 | -0.017 |
| Carnobacteriaceae_unclassified | 0.17043 | 0.61067 | 0 | 0.12 | -0.0253 |
| Sphingomonas | 0.17918 | 0.61067 | 0.043478 | 0.28 | -0.0485 |
| Shuttleworthia | 0.17925 | 0.61067 | 1.7826 | 1.08 | 0.131 |
| Lachnospiraceae_UCG_002 | 0.187 | 0.61067 | 1.0435 | 0.72 | 0.0651 |

* Statistically significant correlations are displayed in orange (no statistically significant geniuses were identified).
