# Supplementary material for: Correlations between the Composition of the Bovine Microbiota and Vitamin B12 Abundance
Source: mSystems. 2020 Mar 3;5(2):e00107-20. doi: 10.1128/mSystems.00107-20 (PMC7055655; doi:10.1128/mSystems.00107-20)
Supplement: TABLE S7 [file mSystems.00107-20-st007.docx]

Table S7 – Samples used to analyse differences in the milk microbiome based total vitamin B12

|  | High concentration of vitamin B12 in plasma and high yield of vitamin B12 in milk | High concentration of vitamin B12 in plasma and low yield of vitamin B12 in milk |
| --- | --- | --- |
| Samples used for Analysis | M4, M7, M12, M14, M15, M16, M18, M19, M25, M26, M27, M30, M37, M61, M67, M71, M72, M73, M77, M79, M80, M90, M93 | M2, M11, M24, M32, M33, M35, M36, M38, M39, M41, M42, M49, M58, M59, M60, M63, M64, M69, M70, M76, M81, M85, M91 |
