# Supplementary material for: Correlations between the Composition of the Bovine Microbiota and Vitamin B12 Abundance
Source: mSystems. 2020 Mar 3;5(2):e00107-20. doi: 10.1128/mSystems.00107-20 (PMC7055655; doi:10.1128/mSystems.00107-20)
Supplement: TABLE S8 [file mSystems.00107-20-st008.docx]

Table S8- LEfSe values for top 50 bacterial taxa at the genus level correlated to vitamin B12 yield in the milk*

| Bacteria Genus | Pvalues | FDR | Gained in Udder | Lost in Udder | LDAscore |
| --- | --- | --- | --- | --- | --- |
| Acetitomaculum | 0.0033429 | 0.62181 | 36.261 | 5.9565 | 1.21 |
| Prevotellaceae_NK3B31_group | 0.009601 | 0.62181 | 9.6087 | 0 | 0.764 |
| Phreatobacter | 0.011494 | 0.62181 | 53.435 | 183.96 | -1.82 |
| Weissella | 0.014652 | 0.62181 | 277.78 | 144.48 | 1.83 |
| Sphingomonas | 0.014742 | 0.62181 | 125.65 | 255.39 | -1.82 |
| Escherichia_Shigella | 0.018328 | 0.62181 | 109.52 | 219.52 | -1.75 |
| Haemophilus | 0.021152 | 0.62181 | 26.435 | 6.4783 | 1.04 |
| Clostridiales_unclassified | 0.023421 | 0.62181 | 36.261 | 2.6957 | 1.25 |
| Ruminococcaceae_UCG_004 | 0.038685 | 0.62181 | 3.9565 | 0 | 0.474 |
| Prevotella_6 | 0.038685 | 0.62181 | 0 | 4.2174 | -0.493 |
| dgA_11_gut_group | 0.038685 | 0.62181 | 4.9565 | 0 | 0.541 |
| Alloprevotella | 0.038685 | 0.62181 | 6.7826 | 0 | 0.643 |
| Oribacterium | 0.041902 | 0.62181 | 2.0435 | 12.652 | -0.8 |
| Ruminobacter | 0.053789 | 0.62181 | 21.87 | 26.174 | -0.499 |
| Tepidimonas | 0.055117 | 0.62181 | 6.1739 | 16.13 | -0.777 |
| Treponema_2 | 0.055715 | 0.62181 | 30.391 | 73.087 | -1.35 |
| Gemella | 0.063522 | 0.62181 | 7.913 | 1 | 0.649 |
| Proteiniphilum | 0.0658 | 0.62181 | 1.7391 | 4.6522 | -0.39 |
| Flaviflexus | 0.068695 | 0.62181 | 0.13043 | 7.2609 | -0.659 |
| Obscuribacterales_ge | 0.070597 | 0.62181 | 1.3043 | 6.8261 | -0.575 |
| Trueperella | 0.074871 | 0.62181 | 443.91 | 4.3043 | 2.34 |
| Prevotellaceae_UCG_003 | 0.076416 | 0.62181 | 0.13043 | 0 | 0.0274 |
| Lachnospiraceae_AC2044_group | 0.076614 | 0.62181 | 0.73913 | 0 | 0.137 |
| Ruminiclostridium_5 | 0.076614 | 0.62181 | 1.3043 | 0 | 0.218 |
| Tyzzerella_3 | 0.076614 | 0.62181 | 0 | 1.913 | -0.291 |
| vadinBE97_ge | 0.076614 | 0.62181 | 2.4348 | 0 | 0.346 |
| Quadrisphaera | 0.076614 | 0.62181 | 0 | 2.4783 | -0.35 |
| Allorhizobium_Neorhizobium_Pararhizobium_Rhizobium | 0.076614 | 0.62181 | 0 | 5.4348 | -0.57 |
| Oxyphotobacteria_unclassified | 0.076614 | 0.62181 | 0 | 7.8696 | -0.693 |
| Nostocales_unclassified | 0.076614 | 0.62181 | 0 | 8.8696 | -0.735 |
| Rubellimicrobium | 0.080923 | 0.62181 | 3.5652 | 0.65217 | 0.39 |
| Staphylococcaceae_unclassified | 0.091268 | 0.62181 | 12.348 | 3.3478 | 0.74 |
| Hymenobacter | 0.094863 | 0.62181 | 8.5217 | 2.3913 | 0.609 |
| Tepidiphilus | 0.096346 | 0.62181 | 0.73913 | 17.348 | -0.969 |
| Bacillaceae_unclassified | 0.096863 | 0.62181 | 15 | 30.652 | -0.946 |
| Blastococcus | 0.10252 | 0.62181 | 5.6087 | 9.6957 | -0.483 |
| uncultured_ge | 0.11583 | 0.62181 | 44.217 | 9.8696 | 1.26 |
| Thermicanus | 0.12413 | 0.62181 | 13.522 | 122.3 | -1.74 |
| Parasutterella | 0.13451 | 0.62181 | 5.4348 | 1.6957 | 0.458 |
| Desulfotomaculum | 0.13783 | 0.62181 | 0.30435 | 3.4348 | -0.409 |
| Dermacoccus | 0.13783 | 0.62181 | 0.26087 | 6.2174 | -0.6 |
| Cellvibrio | 0.13783 | 0.62181 | 0.30435 | 8.6957 | -0.716 |
| Psychrobacter | 0.13783 | 0.62181 | 0.043478 | 14.87 | -0.925 |
| Ruminococcaceae_UCG_005 | 0.13802 | 0.62181 | 392.39 | 430.57 | -1.3 |
| Actinomyces | 0.13897 | 0.62181 | 11.043 | 1.6087 | 0.757 |
| Bifidobacterium | 0.13934 | 0.62181 | 10.826 | 28.913 | -1 |
| Dorea | 0.14448 | 0.62181 | 4.3043 | 7.3913 | -0.405 |
| Prevotellaceae_UCG_004 | 0.14818 | 0.62181 | 11.913 | 3.4348 | 0.719 |
| Coprococcus_3 | 0.14891 | 0.62181 | 0.13043 | 2.1739 | -0.306 |
| Lachnospiraceae_FE2018_group | 0.15276 | 0.62181 | 0.13043 | 0 | 0.0274 |

* Statistically significant correlations are displayed in orange (no statistically significant geniuses were identified).
