# Supplementary material for: Correlations between the Composition of the Bovine Microbiota and Vitamin B12 Abundance
Source: mSystems. 2020 Mar 3;5(2):e00107-20. doi: 10.1128/mSystems.00107-20 (PMC7055655; doi:10.1128/mSystems.00107-20)
Supplement: TEXT S1 [file mSystems.00107-20-s0001.docx]

Supplementary Methods

The following commands were used to process and analyze 16S rRNA taregeted amplicon sequencing data:

>make.contigs(file=stability.files, processors=7)

>summary.seqs(fasta=stability.trim.contigs.fasta, processors=7)

>screen.seqs(fasta=stability.trim.contigs.fasta, group=stability.contigs.groups, summary=stability.trim.contigs.summary, maxambig=0, maxlength=253, minlength=253, processors=7)

>summary.seqs(fasta=stability.trim.contigs.good.fasta, processors=7)

>unique.seqs(fasta=stability.trim.contigs.good.fasta)

>count.seqs(name=stability.trim.contigs.good.names, group=stability.contigs.good.groups)

>summary.seqs(fasta=stability.trim.contigs.good.unique.fasta, count=stability.trim.contigs.good.count_table, processors=7)

>align.seqs(fasta=stability.trim.contigs.good.unique.fasta, reference=silva.v4.fasta, processors=7)

>summary.seqs(fasta=stability.trim.contigs.good.unique.align, count=stability.trim.contigs.good.count_table, processors=7)

>screen.seqs(fasta=stability.trim.contigs.good.unique.align, count=stability.trim.contigs.good.count_table, summary=stability.trim.contigs.good.unique.summary, start=1968, end=11550, maxhomop=8, processors=7)

>summary.seqs(fasta=stability.trim.contigs.good.unique.good.align, count=stability.trim.contigs.good.good.count_table, processors=7)

>filter.seqs(fasta=stability.trim.contigs.good.unique.good.align, vertical=T, trump=., processors=7)

>split.abund(fasta=stability.trim.contigs.good.unique.good.filter.fasta, count=stability.trim.contigs.good.good.count_table, cutoff=20, accnos=true)

>summary.seqs(fasta=stability.trim.contigs.good.unique.good.filter.abund.fasta, count=stability.trim.contigs.good.good.abund.count_table, processors=7)

>unique.seqs(fasta=stability.trim.contigs.good.unique.good.filter.abund.fasta, count=stability.trim.contigs.good.good.abund.count_table)

>pre.cluster(fasta=stability.trim.contigs.good.unique.good.filter.abund.unique.fasta, count=stability.trim.contigs.good.unique.good.filter.abund.count_table, diffs=2, processors=7)

>chimera.uchime(fasta=stability.trim.contigs.good.unique.good.filter.abund.unique.precluster.fasta, count=stability.trim.contigs.good.unique.good.filter.abund.unique.precluster.count_table, dereplicate=t, processors=7)

>remove.seqs(fasta=stability.trim.contigs.good.unique.good.filter.abund.unique.precluster.fasta, accnos=stability.trim.contigs.good.unique.good.filter.abund.unique.precluster.denovo.uchime.accnos)

>summary.seqs(fasta=stability.trim.contigs.good.unique.good.filter.abund.unique.precluster.pick.fasta, count=stability.trim.contigs.good.unique.good.filter.abund.unique.precluster.denovo.uchime.pick.count_table, processors=7)

>classify.seqs(fasta=stability.trim.contigs.good.unique.good.filter.abund.unique.precluster.pick.fasta, count=stability.trim.contigs.good.unique.good.filter.abund.unique.precluster.denovo.uchime.pick.count_table, reference=silva.v4.fasta, taxonomy=silva.nr_v132.tax, cutoff=80, processors=7)

>remove.lineage(fasta=stability.trim.contigs.good.unique.good.filter.abund.unique.precluster.pick.fasta, count=stability.trim.contigs.good.unique.good.filter.abund.unique.precluster.denovo.uchime.pick.count_table, taxonomy=stability.trim.contigs.good.unique.good.filter.abund.unique.precluster.pick.nr_v132.wang.taxonomy, taxon=Mitochondria-Archaea-Eukaryota)

>dist.seqs(fasta=stability.trim.contigs.good.unique.good.filter.abund.unique.precluster.pick.pick.fasta, cutoff=0.10)

>cluster(column=stability.trim.contigs.good.unique.good.filter.unique.precluster.abund.pick.pick.dist, count=stability.trim.contigs.good.unique.good.filter.unique.precluster.abund.denovo.uchime.pick.pick.count_table)

>make.shared(list=stability.trim.contigs.good.unique.good.filter.unique.precluster.abund.pick.pick.an.unique_list.list, count=stability.trim.contigs.good.unique.good.filter.unique.precluster.abund.denovo.uchime.pick.pick.count_table, label=0.03)

>classify.otu(list=stability.trim.contigs.good.unique.good.filter.unique.precluster.abund.pick.pick.an.unique_list.list, count=stability.trim.contigs.good.unique.good.filter.unique.precluster.abund.denovo.uchime.pick.pick.count_table, taxonomy=stability.trim.contigs.good.unique.good.filter.unique.precluster.abund.pick.nr_v132.wang.pick.taxonomy, label=0.03)

>phylotype(taxonomy=stability.trim.contigs.good.unique.good.filter.unique.precluster.abund.pick.nr_v132.wang.pick.taxonomy)

>make.shared(list=stability.trim.contigs.good.unique.good.filter.unique.precluster.abund.pick.nr_v132.wang.pick.tx.list, count=stability.trim.contigs.good.unique.good.filter.unique.precluster.abund.denovo.uchime.pick.pick.count_table, label=1)

>classify.otu(list=stability.trim.contigs.good.unique.good.filter.unique.precluster.abund.pick.nr_v132.wang.pick.tx.list, count=stability.trim.contigs.good.unique.good.filter.unique.precluster.abund.denovo.uchime.pick.pick.count_table, taxonomy=stability.trim.contigs.good.unique.good.filter.unique.precluster.abund.pick.nr_v132.wang.pick.taxonomy, label=1)
